# Telemedicine and Neurology: A Survey of Neurology Patients in a Nigerian Tertiary Hospital

**DOI:** 10.7759/cureus.57916

**Published:** 2024-04-09

**Authors:** Aliu O Yakubu, Oluwakemi Olalude, Mayowa Salami, Augustine C Amuta, Abeedat Amusa, Hasanat A Salaudeen, Ayodeji J Awoyemi

**Affiliations:** 1 Department of Old Age Psychiatry, University Hospital Wishaw NHS Trust, Wishaw, GBR; 2 Department of Internal Medicine, Lagos State University Teaching Hospital, Lagos, NGA; 3 Department of Paediatrics, Princess Royal Maternity Hospital NHS Trust, Glasgow, GBR; 4 Department of Health and Wellness, Prince George's County Health Department, Upper Marlboro, USA; 5 Department of Medicine and Surgery, Obafemi Awolowo College of Health Sciences, Olabisi Onabanjo University, Ago Iwoye, NGA

**Keywords:** telecommunications, telemedicine, remote consultation, developing country, teleneurology

## Abstract

Background

Telemedicine has been recognized as a viable solution for addressing the shortage of medical professionals in developing countries such as Nigeria. Tele-neurology has the potential to provide remote consultations and care for patients with neurological conditions, thereby reducing the burden of travel and improving access to medical care. Despite its growing popularity, there is a lack of research on patient’s views on this mode of care delivery in Nigeria. This study was conducted to investigate patient's perspectives on the use of tele-neurology in Nigeria.

Methodology

A descriptive cross-sectional study was conducted among 398 neurology patients at Olabisi Onabanjo University Teaching Hospital, Sagamu, Ogun State, Nigeria. The data obtained were analyzed using descriptive statistics and a chi-square test using p < 0.05.

Results

Only 3% of our respondents had previously used telemedicine, with 78.1% of the respondents open to using telemedicine as a means of consultation. The disadvantages of telemedicine noted include limitations in assessing neurological status (94.7%), difficulty in explaining health conditions (84.4%), and lack of technical support (14.6%). The majority of respondents (96.5%) believed telemedicine will help in saving time. There was a statistically significant association between propensity to use telemedicine and time spent in the hospital (0.045) and time off work (<0.001). The propensity to use telemedicine was statistically significant to the use of email (0.001) and type of email address (0.001).

Conclusion

The findings suggested that there is a need for healthcare providers and policymakers to invest in developing telemedicine to improve access to care.

## Introduction

The field of telemedicine has become increasingly explored as a means of healthcare delivery, particularly since the advent of the coronavirus disease 2019 (COVID-19) pandemic. Due to the lockdown and the need for social distancing, it was imperative for other means of healthcare delivery to be implemented, whilst maintaining and prioritizing patient’s health. Following the onset of the pandemic, a rapid rise in the use of telemedicine was noted, especially in developed countries. Up to 95% of health centers reported using telemedicine as compared with 43% that previously reported the capability of providing telemedicine prior to the pandemic [1,2].

The American Academy of Family Physicians (AAFP) defines telemedicine as the practice of medicine using technology to deliver care at a distance, over a telecommunications infrastructure, between a patient at an originating (spoke) site and a physician, or other practitioner licensed to practice medicine, at a distant (hub) site [3]. It refers to healthcare delivery or the exchange of health information across distances and is neither a branch of medicine nor a technology in itself [4].

Telemedicine as it pertains to neurological diseases was defined by Chirra et al. as an interface in a virtual patient-physician relationship to provide primary and secondary care in neurodegenerative, cerebrovascular, neuro-oncological, and neuroinflammatory disorders; assist the remote management of deep brain stimulation (DBS), transcranial direct current stimulation (tDCS), and infusion pumps; deliver highly specialized visits (telegenetics), diagnostic consultations (teleradiology and telepathology), or rehabilitative programs (telerehabilitation); and monitor motor and nonmotor functions in an ecologically valid environment (telemetry) [5]. 

The advent of this method of healthcare delivery opened the door to other possibilities and has persisted even beyond the pandemic as a result of its many benefits to both the healthcare provider and the patient. Telemedicine is particularly useful in providing care to patients who live a far distance from the hospital, in the setting of disasters, and public health emergencies [6,7]. Physicians have even reported that telemedicine offered much better quality of healthcare as compared with in-person hospital visits [7]. For patients with chronic diseases who may require long-term follow-up including those with a neurological disease which is our focus, telemedicine is believed to provide great benefits leading to improved outcomes [8].

This method is however still budding in developing countries and needs to be explored even further, as it has great potential for benefit across healthcare delivery, particularly in decreasing the wide healthcare gap. In this study, we have focused on neurological patients, as neurological conditions contribute significantly to the global burden of disease, and were found to be responsible for almost 10 million deaths and about 349 million disability-adjusted life years (DALYs) in 2019 [9]. With the poor neurologist-to-population ratio in Nigeria [10,11], understanding the patient view on telemedicine as a resource, especially in the setting of neurological disorders, would help in tailoring its use to their needs, as these patients often require long-term care, and this could improve the success of this practice.

Although studies on telemedicine and the physician’s perspective abound, there appears to be a paucity of data on the patient’s perspective and experience, particularly in developing countries and sub-Saharan Africa. This study explored the patient’s perspective on the use of telemedicine as it pertains to the field of neurology in Nigeria. Understanding the perception of patients would give better insight into how teleneurology in Nigeria can be structured to encourage its adoption and its benefits to both the provider and the patient.

This article was previously posted to the Research Square preprint server on 19 Feb 2024.

## Materials and methods

Study design and population 

This was a descriptive cross-sectional study conducted among 398 Neurology patients at Olabisi Onabanjo University Teaching Hospital (OOUTH) between June 2023 and October 2023. The OOUTH is a state-owned tertiary institution, formerly Ogun State University Teaching Hospital (OSUTH) located at Sagamu, Ogun State, Southwest Nigeria. The hospital is a prominent tertiary healthcare institution situated in the south-western region of Nigeria, which offers specialized care. The neurology clinic is held weekly at the medicine outpatient department, where participants were recruited for the study. A validated questionnaire developed by Landi et al. [12] was utilized in this study. The questionnaire consisted of 32 questions, categorized into four subsections: 1) Sociodemographic, 2) Access to the hospital, 3) Internet access and use, and 4) Knowledge about telemedicine. A pilot study was conducted among 25 patients to test the clarity of our questionnaire. The questionnaire was modified according to the feedback before the actual data collection.

Ethical consideration

The study protocol was approved by the Ethical Committee of the Institute of Advanced Medical Research and Training, College of Medicine, University of Ibadan, Nigeria (Approval number-UI/UCH/23/0499). 

Inclusion and exclusion criteria 

Neurology patients who have attended the clinic at least once previously were included regardless of their age, gender, race, and socio-economic class. Neurology inpatients and individuals with significant neuro-cognitive decline were excluded from participating in the study.

Sample size determination

The sample size was calculated using the Daniel sample size formula [13]. 

$n= ^{\frac{{{Z^{2}}}P(1-P)}{d^2{}}}$

Where n is the sample size, Z is the statistic corresponding to the level of confidence (1.96 for 95% confidence interval), P is the prevalence of 54%, as reported by Landi et al. [12] indicating the proportion of patients open to using telemedicine. The minimum sample size was 376 participants. 

Data collection 

The simple random sampling technique was employed for the study. The participants were given enough information regarding the study which helped them make informed decisions. A written informed consent was obtained from each participant before administering the questionnaire. A research assistant was recruited to help with the administration of the questionnaire to the respondents at the neurology outpatient clinic. The research assistant ensured the questionnaire was completely filled and provided clarification to respondents when needed.

Data analysis 

The data was sorted with Microsoft Excel and exported into IBM SPSS Statistics for Windows, Version 22 (Released 2013; IBM Corp., Armonk, New York, United States) for statistical analysis. Variables were presented as frequency (N) and percentages (%). Chi-Square was used to determine the association between variables (access to hospital and internet) and propensity to use telemedicine. The level of significance (p-value) was set at 5%.

## Results

A total of 398 patients participated in the survey with 100% response rate, with 52.3% (208) of the respondents being male, and the majority of them (291, 73.1%) were married and Christians (264, 66.3%). One-hundred and eighty-five (46.4%) of respondents were within the 51-70 year age range, and 172 (43.2%) had completed tertiary education. 61.8% (246) were employed, and a majority (96.5%, 384) earned less than 215 US dollars. 84.4% (336) of participants did not require assistance with mobility (Table 1).

**Table 1:** Socio-demographic characteristics of participants The data have been represented as N and %.

Variables	Years	N	%
Age	10 – 30	76	19.1
	31 – 50	94	23.6
	51 – 70	185	46.4
	71 – 90	43	10.8
Gender	Male	208	52.3
	Female	190	47.7
Education	None	6	1.5
	Primary	26	6.5
	Secondary	165	41.5
	Tertiary	172	43.2
	Postgraduate	29	7.3
Religion	Christianity	264	66.3
	Islam	133	33.4
	Traditionalist	1	0.3
Marital status	Single	68	17.1
	Married	291	73.1
	Divorced	16	4
	Widow	23	5.8
Employment status	Employed	246	61.8
	Retired	55	13.8
	Unemployed	97	24.4
Income bracket ($)	0-215	384	96.5
	>215	14	3.5
Require assistance	No	336	84.4
	Yes, bilateral assistance	5	1.3
	Yes, I use a wheelchair	8	2
	Yes, unilateral assistance	49	12.3

One hundred and ninety-seven out of 398 (49.5%) spent less than an hour to reach the hospital, while 45.2% (180) spent 5 to 6 hours within the facility during clinic visits. More than half of the participants (64.1%, 255) required an accompanying person during their visits to the hospital. More than half of the participants (65.8%, 262) do not require time off from work (Table 2).

**Table 2:** Access to the hospital The data have been represented as N and %.

Variables	Responses	N	%
Time required to get to the health facility	<1 hour	197	49.5
	1- 2 hours	180	45.2
	3 – 4 hours	21	5.3
Time spent in the hospital	1- 2 hours	20	5
	3- 4 hours	153	38.4
	5- 6 hours	180	45.2
	>6 hours	45	11.3
Employer permission for health facility visit/Time off work	No	262	65.8
	Not working	2	0.5
	Yes, a day	119	29.9
	Yes, less than a day	11	2.8
	Yes, more than a day	4	1
Accompanying person	Yes	255	64.1
	No	90	22.6
	Sometimes	53	13.3
Means of transportation	Motorcycle	77	19.4
	Private car	69	17.3
	Public buses	252	63.3

87.7% (349) of the participants have used the internet, and 42.7% (170) utilized it for medical purposes. The majority (95.7%,381) had smartphones, and 336 (84.4%) had personal email addresses. Video/social media platforms popular among the participants were WhatsApp (91.2%, 363), followed by Zoom (21.9%, 87) and Google Meet (16.3%, 65) as shown in Table 3.

**Table 3:** Internet access and use The data have been represented as N and %.

Variables	Responses	N	(%)
Use the internet	Yes	349	87.7
	No	49	12.3
Use a digital device (preference)	None	6	1.5
	Personal computer	5	1.2
	Smart phone	381	95.7
	Tablet	6	1.6
Use the internet for medical reasons	No	228	57.3
	Yes	170	42.7
Use the email	Yes	306	76.9
	No	92	23.1
Type of email address	Friends	5	1.3
	Personal	336	84.4
	Relative	21	5.3
	Work	7	1.8
	None	29	7.2
Video/social media platform (Multiple entries)	Whatsapp	363	91.2
	Zoom	87	21.9
	Google meet	65	16.3
	Teams	42	10.6
	FaceTime	7	1.8
	Skype	13	3.3
	None	36	9

Only 12 out of 398 (3%) respondents had previous experience with telemedicine, and only 5% (20) had heard about it before. The majority of the respondents (78.1%, 311) were open to using telemedicine as a means of consultation. More than half of the respondents (56.5%, 225) preferred teleconsultation to telephone calls. Disadvantages of telemedicine noted included limitations in assessing neurological status (94.7%, 377), difficulty in explaining health problems (84.4%, 336), and lack of technical support (14.6%, 58). 96.5% (384) believed that telemedicine would help save time. Most participants (76.4%, 304) were open to upload their personal information on the Internet. Only 13.8% (55) believed telemedicine was complete. Situations in which respondents believed telemedicine could replace hospital visitation included living far from the hospital (96.5%, 384), patients with mobility issues (93%, 370), medication change (27.1%, 108), and emergency situations (16.8%, 67) (Table 4).

**Table 4:** Knowledge about telemedicine The data have been represented as N and %.

Variables	Responses	N	100%
Heard of telemedicine	Yes	20	5
	No	378	95
Previous telemedicine experience	Yes	12	3
	No	386	97
Evaluation of previous telemedicine experience if any	Some connection problems but solved	8	66.8
	No connection problems	2	16.6
	Assistance needed	2	16.6
Open to using telemedicine	No, not interested	87	21.9
	Yes, I would like to	311	78.1
Advantages of telemedicine (multiple entries)	No accompanying person needed	101	25.4
	Saves money	311	78.1
	Saves time	384	96.5
Disadvantages of telemedicine (multiple entries)	Difficult to explain medical/health problems	336	84.4
	No possibility of assessing neurological status	377	94.7
	Difficult to use	20	5
	Unreliable	44	11.1
	Lack of technological support	58	14.6
	It is expensive	1	0.3
Telemedicine is complete	Agree	55	13.8
	Disagree	78	19.6
	Neutral	265	66.6
Telemedicine is preferable over the telephone	Yes	225	56.5
	No/not sure	173	43.5
Situations where telemedicine can replace center visit (multiple entries)	Evaluation of medication change	108	27.1
	Multidisciplinary counseling	41	10.3
	Patients with mobility issue	370	93
	Patients from far places	384	96.5
	Evaluation of new symptoms	8	2
	In emergency situations	67	16.8
Open to having your personal information on the internet	Yes	304	76.4
	No	52	13.1
	Not sure	42	10.5

There was a statistically significant association between propensity to use telemedicine and time off work (<0.001). Average time spent in the hospital had a statistically significant association with propensity to use telemedicine (p-0.045) (Table 5).

**Table 5:** Association between access to the hospital and open to use telemedicine The data have been represented as N and %.

Variables	Responses	Open to use telemedicine, n (%)	Not open to use of telemedicine, n (%)	p-value
Time required to get to the health facility	<1 hour	39 (19.8)	158 (80.2)	0.072
	1 -2 hours	40 (22.2)	140 (77.8)	
	3- 4 hours	8 (38.1)	13 (61.9)	
Time spent in the hospital	1- 2 hours	4 (20.0)	16 (80.0)	0.045
	3- 4 hours	29 (19.0)	124 (81.0)	
	5- 6 hours	37 (20.6)	143 (79.4)	
	>6 hours	17 (37.8)	28 (62.2)	
Employer permission for health facility visit/Time off work	No	74 (28.2)	188 (71.8)	<0.001
	Not working	0 (0.0)	2 (100.0)	
	Yes, a day	13 (10.9)	106 (89.1)	
	Yes, less than a day	0 (0.0)	11 (100.0)	
	Yes, more than a day	0 (0.0)	4 (100.0)	
Accompanying person	Yes	62 (24.3)	193 (75.7)	0.097
	No	17 (18.9)	73 (81.1)	
	Sometimes	8 (15.1)	45 (84.9)	
Means of transportation	Motorcycle	6 (7.8)	71 (92.2)	0.433
	Private car	18 (26.1)	51 (73.9)	
	Public buses	63 (25.0)	189 (75.0)	

The propensity to use telemedicine was statistically significant to the use of email (0.001) and type of email address (0.001) (Table 6).

**Table 6:** Association between access to the Internet and being open to use telemedicine The data have been represented as N and %.

Variables	Responses	Not open to use telemedicine n (%)	Open to use telemedicine n (%)	p-value
Use the internet	Yes	76 (20.7)	291 (79.3)	0.056
	No	11 (35.5)	20 (64.5)	
Use a digital device (preference)	None	3 (50.0)	3 (50.0)	0.083
	Personal computer	2 (40.0)	3 (60.0)	
	Smart phone	81 (21.3)	300 (78.7)	
	Tablet	1 (17.0)	5 (83.0)	
Use the internet for medical reasons	Yes	21 (12.4)	149 (87.6)	0.739
	No	66 (28.9)	162 (71.1)	
Use the email	Yes	49 (16.0)	257 (84.0)	<0.001
	No	38 (41.3)	54 (58.7)	
Type of email address	Friends	0 (0.0)	5 (100.0)	<0.001
	Personal	61 (18.2)	275 (81.8)	
	Relatives	3 (14.3)	18 (85.7)	
	Work	0 (0.0)	6 (100.0)	
	Personal and work	0 (0.0)	1 (100.0)	
	None	23 (79.3)	6 (20.7)	

## Discussion

Telemedicine refers to healthcare delivery that entails the exchange of health information across distances and it is neither a branch of medicine nor a technology in itself [4]. It is an emerging means of the practice of medicine with a lot of potential to decrease the healthcare gap in neglected areas. In this study, we have focused on neurological patients as it is important to assess their perceptions and identify potential factors that may impact the utilization of telemedicine.

Nearly all participants (98.5%, 392) had at least a primary school education. The level of education has been shown to influence the use of telemedicine as patients with tertiary education are likely to use telemedicine, [14], with more than half of our participants (50.5%, 201) having tertiary education, this must have influenced their high level of openness to adopt telemedicine, as noted in our study. More than 90% (384) of our respondents earned less than 215 USD monthly, this is in contrast to a study in which 50.1% of respondents were high-medium income earners with no association with the adoption of telemedicine [14]. This might have accounted for many of our respondents who were open to the use of telemedicine as it is an affordable means of healthcare. Although it took the majority of respondents (94.7%, 377) two hours or less to get to the hospital for their health visit, 95% (378) spent three hours or more in the hospital during each health visit. This large amount of time spent at each visit could easily lead to fatigue, impede a patient's work and productivity, and discourage attendance at future visits. This cascade could be a determining factor for considering telemedicine usage.

29.9% (119) of patients were given a day off work for health visits compared to 65.8% (262) who weren’t granted permission off duty for hospital visits. It is not surprising that the majority of patients (96.5%, 384) believed that telemedicine would help in saving time spent accessing healthcare. Our study found an association between openness to use telemedicine and hours spent in the hospital, and time off at work. This is in contrast with a study among patients with multiple sclerosis in that those factors did not influence the propensity to use telemedicine. Patients in other countries who have utilized telemedicine have attested to its cost-effectiveness, time-saving, and convenience [15,16]. It is also helpful in reaching patients in rural areas who may not have access to medical care and ultimately reduces rural-urban health disparities [17]. Patients can save valuable time, while healthcare providers can optimize their schedules by allotting specific time slots to patients and enhancing overall efficiency in patient care delivery. Neurologists in Nigeria have also acknowledged telemedicine as a viable strategy to bridge the healthcare disparity and enhance the availability of high-quality medical services [18].

Our study showed that 84.4% (336) of patients did not require mobility aids but more than half of the participants did require an accompanying person. These findings are consistent with a previous study among patients with multiple sclerosis in which 75% of the patients did not require mobility assistance [12]. Although telemedicine is believed to assist healthcare delivery, especially for patients with varying degrees of mobility impairment [15], both studies didn't find a significant correlation between the propensity to use telemedicine and the patient's level of disability. More than half of our participants (63.3%, 252) used public transport, and though our study did not find an association between openness to telemedicine and means of transport, telehealth has been shown to address transport barriers. In a study in the US, 41% of respondents experienced transport problems that led to missed and rescheduled appointments, and 78.3% of telehealth users found it easier to access medical care without the need to travel [19].

As it is a digital age, it is unsurprising that almost all participants in this study (98.5%, 392) possessed a mobile device or personal computer, with nearly 90% having internet access. These figures align with a separate study indicating that 97% of African American parents own cell phones, and 80% have internet access [20]. Given the rising use of mobile health technologies in Nigeria, [21] the widespread availability of mobile devices, good internet connectivity, and familiarity with online platforms are pivotal for advancing and embracing teleneurology in the region. Notably, our study revealed no significant association between internet access and a willingness to explore telemedicine.

Our findings demonstrated that over three-quarters (76.9%, 306) of respondents were acquainted with electronic mails, and 84.4%(336) had personal email addresses. Our results also showed that the use of email and type of email address was associated with a higher propensity to use telemedicine. Email address has been shown to help provide access to medical treatment, improve follow-up and adherence to treatment [22]. Email addresses are used as a primary means of consultation and tele-diagnosis in successful telemedicine projects [23]. Our findings suggested that the majority of our participants were familiar with digital platforms such as WhatsApp, Zoom, Google Meet, and FaceTime. These platforms are suitable platforms for telemedicine and means of communication.

Even though only 3% (12) of the respondents had prior experience with telemedicine, and only about 5% (20) had heard about it before the study, the majority (78.1%, 311) expressed openness to televisitations. This emphasizes the existing user gap and highlights the potential for its adoption among patients. This study serves as a crucial step in addressing the understanding of tele-neurology and acceptance rates among patients in Nigeria. Our finding is similar to another study in India, where 82.6% of participants were initially unaware of telemedicine, but their attitude shifted positively once they gained a better understanding of the technology [24]. Another study in Italy reported a slightly lower percentage (54%) of multiple sclerosis patients being open to telemedicine visits. Importantly, a larger percentage of their patients (43%) were already aware of telemedicine even before the COVID-19 pandemic. This disparity shows the knowledge gap and health disparity between low-middle-income countries and high-income countries [12].

The consensus among nearly all participants in our study regarding the benefits of telemedicine use was centered on its time and cost-saving advantages. Of the participants with prior experience, 83.4% (10) reported encountering connection issues and assistance during usage, although these were deemed resolvable. The remaining participants were evenly divided between those requiring assistance to address the problems and those who encountered no connection issues at all. Poor internet connectivity has been recognized as a barrier to the use of telemedicine in Nigeria [18]. Our study also identified concerns about the limitation of telemedicine in assessing neurological status as a significant drawback. Other challenges included difficulty in articulating health problems and a lack of technical support. These findings align with a multi-country study highlighting technical challenges and the absence of physical examination as major hurdles to virtual visits [16]. Physicians have also acknowledged these limitations, particularly the absence of physical examination, as a challenge to reaching an accurate diagnosis with telemedicine [7]. These limitations stress the need for ongoing modification and adaptation of telemedicine strategies.

Furthermore, the study explored potential applications of telemedicine, with a substantial number of respondents recognizing its effectiveness for individuals facing mobility challenges, such as those living with disabilities and the elderly. Telemedicine, by offering virtual consultations, addresses this obstacle. Respondents also identified additional scenarios where telemedicine could be beneficial, particularly for individuals residing at long distances who endure extensive journeys to access healthcare. This suggests that individuals facing such challenges would be open to considering telemedicine if it becomes readily available.

Limitations 

The study's limitations included recall bias, a recognized limitation of cross-sectional studies. Our sample size is modest and restricted to a single center. A multicenter study across Nigeria and Africa would offer a broader understanding of patient perspective on teleneurology, especially considering its early phase of adoption in Africa.

## Conclusions

Teleneurology presents a feasible means of healthcare delivery that should be explored in low-middle-income countries. These should be complementary tools to traditional hospital visits, rather than a complete replacement. Telemedicine has the potential to save time and reach patients who reside far from healthcare facilities, especially those in rural areas who may lack access to specialized care. This is particularly relevant considering the limited ratio of neurologists to the population in countries such as Nigeria. Telemedicine should prioritize the needs of both healthcare providers and patients and implement a feedback mechanism that helps to identify and address the challenges, allowing for necessary adjustment and improvement. Also, a regulatory body dedicated to overseeing telemedicine usage is crucial to maintaining high standards of care and maximizing its benefits.

While there are challenges such as technological barriers, lack of supportive policies, and biases among patients and healthcare personnel in sub-Saharan Africa, we remain confident that these challenges can be overcome, and with the right strategies and implementation, telemedicine can thrive in Nigeria and the entire African continent.
